# Lipidomic Characterization of Muscle and Head of *Litopenaeus vannamei* Exposed to *Lactococcus lactis* D1813 at Varied Levels of Salinity and Dissolved Oxygen

**DOI:** 10.1002/fsn3.71141

**Published:** 2025-10-29

**Authors:** Muhammad Adil, Xinglong Xiao, Muhammad Waseem, Muhammad Talha Afraz, Muhammad Rizwan Javed, Basim M. Alohali, Isam A. Mohamed Ahmed, Muhammad Faisal Manzoor, Felix Kwashie Madilo

**Affiliations:** ^1^ School of Food Science and Engineering South China University of Technology Guangzhou China; ^2^ Department of Food Science & Technology, Faculty of Agriculture & Environment The Islamia University of Bahawalpur Punjab Pakistan; ^3^ Department of Food Science and Nutrition, College of Food and Agricultural Sciences King Saud University Riyadh Saudi Arabia; ^4^ Guangdong Provincial Key Laboratory of Intelligent Food Manufacturing, School of Food Science and Engineering Foshan University Foshan China; ^5^ Faculty of Sciences and Technology ILMA University Karachi Pakistan; ^6^ Food Science and Technology Department Ho Technical University Ho Ghana

**Keywords:** aquaculture, KEGG, lipid metabolism, metabolites, probiotics, shrimps

## Abstract

The present study examined the impacts of *Lactococcus lactis* D1813, salinity (8 and 25 ppt), and dissolved oxygen (DO) concentrations of 8.5 and 3.5 mg/L on *Litopenaeus vannamei* muscle and head lipidomic profile. The muscle and head were examined using liquid chromatography‐mass spectrometry (LC–MS/MS) for lipidomic nutrient profiling. The lipidomic profiling revealed the primary nutritional metabolites in *L. vannamei* muscle and head, which are composed of glycerophospholipids, sphingolipids, sterols, and saturated fatty acids. Huang head had the most glycerophospholipids and sterols, including PC (12:0/18:2), PC (18:0/20:4), PC (16:1/22:6), PC (10:1e/20:4), PC (33:0/18:2), Cer (d14:1/20:0), SM (t18:1/22:4), and SM (d18:0/16:1). Conversely, the T3BS head group had a much greater proportion of sphingolipids and saturated fatty acids, including Cer (d14:1/20:0), SM (t18:1/22:4), SM (d18:0/16:1), SM (d18:1/18:3), and butyric acid, stearic acid, myristic acid, arachidic acid, and lauric acid (*p* < 0.05). The Kyoto Encyclopedia of Genes and Genomes (KEGG) pathway analysis indicated the significant enrichment of glycerophospholipid metabolism, sphingolipid metabolism, glycerolipid metabolism, linolenic acid metabolism, and alpha‐linolenic acid metabolism in T3BS muscle and head. The findings unequivocally demonstrate that the supplement of *L. lactis* D1813 under 25 ppt salinity and 3.5 mg/L DO levels significantly raises the content of sphingolipids and saturated fatty acids and enriches the metabolic pathways of linolenic acid and alpha‐linolenic acid in *L. vannamei* . The results indicated that *L. lactis* D1813 could be used as an effective probiotic to enhance shrimp tolerance and nutritional quality in aquaculture systems under environmental stress conditions. Also, a more desirable lipid profile renders them beneficial to human health, particularly cardiovascular health and brain function. Additional research can be done to assess the therapeutic advantages of *L. vannamei* ‐derived lipids in humans regarding cardiovascular health, cancer, and brain functioning.

## Graphical abstract

Lipidomic characterization of muscle and head of *L. vannamei* . Huang head group showed the highest abundance of glycerophospholipids and sterols. Kyoto Encyclopedia of Genes and Genomes (KEGG) pathway analysis showed significant enrichment in glycerophospholipid, sphingolipids, glycerolipids, linolenic, and alpha‐linolenic acids metabolism in the T3BS muscle and head.

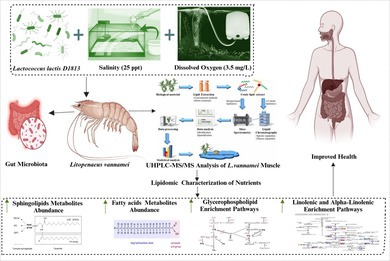

## 1 Introduction

Shrimp ( *Litopenaeus vannamei* ) is a valuable aquatic animal in global aquaculture, characterized by a fast growth rate, broad environmental adaptability in diverse farming conditions, and high nutritional value. *L. vannamei* is cultured by farmers in 47 nations whose total yearly production globally amounts to 6.82 million tons. The major *L. vannamei* ‐producing nations are China, India, Ecuador, Indonesia, Vietnam, Thailand, Mexico, and Brazil. They produce over 100,000 tons annually, with China leading the pack at 2.09 million tons (Nguyen et al. 2019). The essential amino acids, low saturated fats, vitamins (cobalamin, niacin, tocopherol, and riboflavin), and minerals (selenium, magnesium, copper, iodine, phosphorus, and zinc) are all abundant in *L. vannamei* (Hulefeld et al. 2018). According to *L. vannamei* nutritional composition, it contains astaxanthins, 32% polyunsaturated fatty acids (PUFA), specifically ω‐3PUFA (64%), ω‐6PUFA (33%), 10%–20% total acylglycerol, and 65%–70% phospholipids (Li et al. 2023; Salim et al. 2025). These biologically active compounds anticipate several health‐promoting features against thyroid problems, cancer, oxidative stress, cardiovascular risks, blood pressure, and immune dysfunctions (Guo et al. 2024). Lipids are macromolecules and have a variety of vital functions, such as energy reserves, regulation of hormones, maintenance of cellular membrane integrity, and signal transduction. Eicosapentaenoic acid and Docosahexaenoic acid are essential lipids naturally found in *L. vannamei* . Among other essential fatty acids found in *L. vannamei* are sterols, conditionally essential and long‐chain polyunsaturated fatty acids, which are linked to play significant roles in improving metabolism, relieving cardiovascular issues, decreasing inflammation, enhancing cognitive abilities, and improving skin health (Zhang et al. 2022). Shrimp fatty acids are essential for growth, development, cell division, tissue repair, and responses to environmental stress (Emerenciano et al. 2022). Lipidomics provides excellent information on energy metabolism, membrane dynamics, and stress responses. Lipidomic techniques have been used to investigate the degradation processes of lipids in aquatic animals under various environmental conditions, such as salinity, dissolved oxygen, pH, and acidity. This research seeks to enhance the lipid yield and nutritional value (Huang et al. 2019).

The probiotic strain *L. lactis* D1813 is a well‐studied (Pan 2019) and is known to improve gut health, strengthen the immune system, and enhance metabolism in aquatic animals (Cano‐Lozano et al. 2022; Susalam et al. 2024). DO and salinity are two of the most critical water quality parameters in *L. vannamei* culture with a noticeable effect on growth, survival, and overall health (Cano‐Lozano et al. 2022; Hamzah et al. 2024). Some *Bacillus* strains are not yet GRAS (Generally Regarded as Safe) (Elshaghabee et al. 2017). *L. lactis* is classified as Generally Recognized As Safe (GRAS), which makes it more acceptable to consumers and not subject to food control (Kazou 2022). Meanwhile, research was conducted to identify other Vibrio strains phylogenetically associated with the most prevalent disease. Also, horizontal gene transfer of multidrug resistance genes is widespread in Vibrio species (Miller et al. 2021). The role of probiotics has been shown in a recent report to improve lipid metabolism in *L. vannamei* . Probiotics have been shown to enhance the absorption of nutrients and lipid digestion by promoting the production of bile acids and lipases, which break down complex lipids into readily absorbable components (Awaluddin et al. 2025; Goh et al. 2023). Probiotics influence lipid biosynthesis by secreting omega‐3 and omega‐6 fatty acids, which are important in energy production, membrane fluidity, and immunity (Chen et al. 2020). Probiotics, that is, *L. lactis*, enhance intestinal health by significantly altering the microbiota, reducing oxidative stress, and enhancing the effectiveness of nutrient absorption (Ringø et al. 2022; Srifani et al. 2023). Salinity plays a significant role in the lipid metabolism of shrimps. Elevated salinity has the potential to disrupt ionic gradients, leading to oxidative stress and enhancing lipid stability and metabolic pathways (Wang et al. 2024). Likewise, low salinity (> 5 ppt) can compromise osmoregulation and alter membrane lipid composition, restricting the shrimp's ability to efficiently utilize dietary lipids (Lu et al. 2023). Ideal salinity assists in maintaining cellular balance and membrane stability for efficient lipid metabolism and nutrient uptake (Zhao et al. 2022). High DO levels are important in regulating lipid metabolism and energy production (Guo et al. 2024). Similarly, excessively higher levels of DO (i.e., hyperoxia > 8 mg/L) can lead to oxidative stress, which causes lipid peroxidation and disrupts cellular functions, which demands maintaining an appropriate level of DO to support lipid homeostasis, energy balance, and the overall health of shrimp (Nguyen et al. 2022).

Plenty of data is present in the literature, anticipating the nutritional profile, impact of health‐related and environmental factors on *L. vannamei*, and probiotic effect on the health of *L. vannamei* ; in the literature, there is a notable gap showing the combined effect of *L. lactis* D1813 supplementation in addition to different levels of salinity and DO, especially on lipidomic characterization of metabolites. Against this backdrop, this research targeted lipidomic characterization of the muscle and head of *L. vannamei* subjected to *L. lactis* D1813 under varying salinity and DO levels.

## 2 Materials and Methods

### 2.1 Materials, Chemicals and Reagents

Fresh and healthy *L. vannamei* samples (250 of each group, with an average weight of 23 g each) were collected from local farms in Hunan Province, China, and kept in ice bags at 0°C to keep them fresh. The chemicals and reagents are ethanol, methanol, acetonitrile, chloroform, isopropanol, formic acid, hydrochloric acid, hexanoic acid, ammonium acetate and sodium hydroxide. Solvents and reagents (acetonitrile, methanol, ethanol, chloroform, isopropanol, hexane, formic acid, hydrochloric acid, sodium hydroxide, acetic acid and ammonium acetate) were analytical grade (≥ 99% purity) and acquired from Fisher Chemicals (Saint Louis, MO, USA). The probiotic strains of *L. lactis* D1813 were acquired from Symbiotic Biotechnology Co. Ltd., Beijing, China.

### 2.2 Experimental Diet and *L. lactis* D1813 Inoculation

The experimental diet of *L. vannamei* is shown in Table 1. All dry ingredients were carefully measured and combined. Distilled water (410 mL/kg) was added to the dry mix, and gelatin (40 g/kg) served as a binder (Valdez‐Chavez et al. 2024). The mixture was then processed through a meat grinder fitted with a die of 1.2 mm diameter. The resulting wet strings were allowed to dry at room temperature for 24 h. Once dried, the diets were broken down into appropriately sized pellets. They were stored at −20°C until it was time to feed. The proximate analysis was performed following AOAC methods (AOAC 1992). The *L. lactis* D1813 inoculation followed the standard protocol described by Liu et al. (2022). A precisely measured 0.01 g/L of *L. lactis* D1813 was added to the rearing water of shrimps based on the tank volume twice a day, along with the live feeds (rotifers, i.e., *Brachionus plicatilis* , enriched with microalgae species *Nannochloropsis* and *Isochrysis*) 15 min before feeding the shrimps. The mixture was then gently aerated by a fine bubble diffuser (Aqua Air 8000, Saint Louis, MO, USA, 8‐inch diameter, producing bubbles of 20–100 μm) to ensure uniform distribution of the inoculum and allowed to rest for a few minutes. The final volume of *L. lactis* D1813 in the water was adjusted to 10^6^ CFU/mL. This process was repeated twice a day to maintain consistent probiotics (i.e., *L. lactis* D1813) exposures.

**TABLE 1:** Relative abundance of sterols metabolites.

Sterols	HMS	WMS	T3MS	HHS	WHS	T3HS
ChE (0:0)	1.37 ± 0.23^a^	1.38 ± 0.23^a^	1.21 ± 0.03^b^	8.84 ± 0.06^a^	8.25 ± 0.03^b^	8.00 ± 0.01^c^
ChE (22:6)	1.12 ± 0.21^a^	1.13 ± 0.21^a^	1.16 ± 0.21^a^	8.97 ± 0.47^a^	8.06 ± 0.57^b^	7.85 ± 0.03^c^
ST (m20:0/16:1)	1.31 ± 0.10^c^	1.63 ± 1.15^a^	1.51 ± 0.10^b^	8.94 ± 0.02^a^	7.95 ± 0.22^b^	8.03 ± 0.23^b^
ZyE (0:0)	1.11 ± 0.07^a^	1.09 ± 0.07^a^	1.13 ± 0.07^a^	8.70 ± 0.44^a^	7.86 ± 0.49^c^	8.13 ± 0.51^b^
ChE (18:2)	0.95 ± 0.01^a^	0.96 ± 0.01^a^	0.96 ± 0.01^a^	6.30 ± 0.24^a^	6.20 ± 0.98^ab^	6.87 ± 0.06^b^
ChE (20: 5)	0.30 ± 0.01^a^	0.32 ± 0.01^a^	0.33 ± 0.01^a^	6.68 ± 0.27^c^	7.31 ± 0.70^a^	7.15 ± 0.02^b^

*Note:* HMS = Huang muscle sample, WMS = Wei muscle sample, T3MS = T3BS muscle sample. Results are presented as means ± SD and *n* = 3. Values with different alphabets differ significantly (*p* < 0.05) among HMS, WMS, and T3MS. HHS = Huang head sample, WHS = Wei head sample, T3HS = T3BS head sample. Results are presented as means ± SD and *n* = 3. Values with different alphabets differ significantly (*p* < 0.05) among HHS, WHS, and T3HS.

### 2.3 Study Design

The *L. vannamei* samples inoculated with *L. lactis* D1813 were reared for 1 week before being divided into three experimental groups. Each group consisted of thirty selected *L. vannamei*, which were acclimatized to the following experimental conditions: Huang (DO levels 8.5 ± 0.5 mg/L, salinity levels 8 ± 0.1 ppt), T3BS (DO levels 3.5 ± 0.5 mg/L, salinity levels 25 ± 0.1 ppt), and the Wei group is a freshwater *L. vannamei* reared in natural freshwater conditions (0 ppt salinity, ~7.5 mg/L DO) without probiotic supplementation. All experimental groups were acclimatized to their respective salinity and DO conditions for 3 weeks using the same protocol (Jaffer et al. 2020). The experiment was conducted for 7 weeks in 90‐L white plastic tanks with complete aeration systems to maintain stable DO levels and homogeneous water circulation. Water temperature for every group, including the control group (Wei), was maintained at 26°C ± 2°C throughout the experiment. DO levels were maintained at 8.5 ± 0.5, 3.5 ± 0.5, and 7.5 ± 0.5 mg/L for the Huang, T3BS, and Wei groups. *L. vannamei* was provided free access to feed twice daily (at 9:00 and 17:00) and water ad libitum; water was replaced every 5 h, with approximately 20% of the tank volume exchanged each time to maintain water quality and ensure optimal DO levels.

The brine was continuously aerated every 24 h. Before the experiment, the feeding of *L. vannamei* was stopped 24 h in advance, and thirty *L. vannamei* were randomly selected from each group without exoskeleton (*n* = 30) for analysis. Separate samples from the muscle and head were used to ensure the precise determination of the specific composition of each part. Three *L. vannamei* were used for lipidomic analysis, and each parameter was tested in triplicate (*n* = 3). The study adheres to ethical guidelines under protocol no. L20090101. The muscle and head of *L. vannamei* without exoskeletons were immediately flash frozen at −196°C, then stored in an ultra‐freezer (Thermo Scientific ULT1786‐3‐V40) at −80°C ± 1°C for subsequent analysis.

### 2.4 Lipid Extraction

A precisely measured 50 mg sample was weighed into 2‐mL plastic microtubes and mixed with 280 μL of a methanol: water (2:5, v/v) solution and 400 μL of MTBE. Before extraction, a 6‐mm grinding bead was included in each sample. Samples were homogenized at −10°C utilizing a High Throughput Tissue Crusher Wonbio‐96c (Shanghai Wonbio Technology Co. Ltd), functioning at a frequency of 50 Hz for 6 min, followed by sonication at 40 kHz for 30 min at 5°C. The samples were kept at −20°C for 30 min and then subjected to centrifugation at 13,000 *g* at 4°C for 15 min. 350 μL of lipid extracts from the upper phase were transferred to new tubes and evaporated to dryness utilizing a mild nitrogen stream. For Ultra‐High‐Performance Liquid Chromatography‐Mass Spectrometry/Mass Spectrometry (UHPLC–MS/MS) analysis, the samples were reconstituted in 100 μL of a loading solution consisting of isopropanol and acetonitrile (1:1, v/v) via brief sonication in a 5°C water bath. Lipids were centrifuged for 15 min at 13,000 *g* and 4°C using a benchtop centrifuge (Eppendorf 5417R, Eppendorf, Germany). The cleared supernatant was transferred to sample vials, and 2 μL aliquots of each sample were injected into the UHPLC–MS/MS system. Calibration curves for each lipid class were set using external standards within a concentration range of 1–1000 μM to enable precise lipid quantification. Internal standards C17:0 and C20:0 were employed for lipid quantification, with calibration standards created at five distinct concentrations. The detection limits varied from 0.01 to 0.1 μM, while the limits of quantification ranged from 0.1 to 1 μM, ascertained using triplicate analysis of calibration standards. The values were derived using the blank's standard deviation and the calibration curve's slope.

### 2.5 Quality Control (QC) Sample

The quality control method entailed the preparation of QC samples by combining equal volumes of all analytical samples during the system conditioning phase. The QC samples underwent processes and analytical methodologies identical to those of the analytical samples. The recurrent injection of QC samples accurately reflected the entire dataset steadily, as analysis monitoring was conducted after every 10 analytical samples.

### 2.6 UHPLC–MS/MS Analysis

The analysis was carried out at Majorbio Bio‐Pharm Technology Co. Ltd. in Shanghai, China, using a quadrupole Exactive high field mass spectrometer (UHPLC‐Q Exactive HF‐X, Thermo Fisher Scientific) coupled with a C30 column (100 mm × 2.1 mm i.d., 2.6 μm; Thermo, USA) for the liquid chromatography‐mass spectrometry (LC–MS/MS) analysis. One chromatographic solution (solvent A) had 10 mM ammonium acetate in acetonitrile: water (1:1, v/v) with 0.1% (v/v) formic acid, while the other contained 2 mM ammonium acetate in acetonitrile: isopropanol: water (10:88:2, v/v/v) with 0.02% (v/v) formic acid. These solutions made up the mobile phase. A 2 μL volume was injected at a rate of 0.4 mL/min at 40°C for 20 min as the experimental settings for the standard sample injection. All samples must be kept at 4°C due to the analytical time. A benchtop Orbitrap mass spectrometer with a heated electrospray ionization (HESI) source that could operate in positive and negative ion modes was used; the instrument was a Thermo UHPLC Q‐Exactive HF‐X. Parameters of the device included a sheath gas flow pressure of 60 psi, an auxiliary gas flow pressure of 20 psi, an auxiliary gas heater temperature of 370°C, and an ISVF of −3000 V in negative mode and +3000 V in positive mode. Using a normalized impact energy ranging from 20, 40, and 60 V, the DDA method was used for data capture to detect mass values between 200 and 2000 *m*/*z*.

### 2.7 Data Preprocessing and Annotation

The UPLC‐MS/MS data were uploaded into Lipid Search (Thermo, CA) to identify, align, and detect peaks. The lipids were identified by examining their MS/MS fragments, which allowed for a precursor and fragment ion tolerance of 10 ppm. We used the A, B, C, and D grading criteria to evaluate the quality of lipid identification, and we set the m‐score cutoff at 2.0. The data matrix created during the preprocessing phase includes lipid class, retention time (RT), mass‐to‐charge ratio (*m*/*z*), and peak intensity values. The distribution of lipid metabolites among treatment groups was shown by the peak intensities of lipid species, which varied from around 1.0 × 10^5^ to 5.0 × 10^7^ arbitrary units. We used the Majorbio Cloud Platform (cloud.majorbio.com) to analyze the data. We removed variables from the quality control samples if their RSD was more than 30%. The data were transformed using a log^10^ operation to produce the final matrix, which was then used for additional analysis.

### 2.8 Lipidomic Data Analysis

The raw data processing utilized QI software version 2.0 (Waters), which executed peak picking, alignment, and normalization. The EZinfo software version 3.0 from Waters performed principal component analysis (PCA) and partial least‐squares discriminant analysis (PLS‐DA). The significance of metabolites within the *L. vannamei* groups was determined by VIP scores over 1 and *p* values below 0.05, as assessed using one‐way analysis of variance (Olkowicz et al. 2024). The identification of metabolites was conducted using three databases: the Human Metabolome Database (https://www.hmdb.ca/), METLIN (https://metlin.scripps.edu/), and MONA (https://mona.fiehnlab.ucdavis.edu/). The precursor ion comparison necessitated a 10‐ppm error tolerance, but a 20‐ppm error tolerance was permissible for fragment ion analyses. The analysis employed Metabo Analyst (version 5.0) (https://www.metaboanalyst.ca/) to generate heat maps and examine metabolic pathways. The platform analyzed metabolite concentrations by dividing peak areas from integrated processes into the total ion chromatogram contents from sample files (Lai et al. 2021).

### 2.9 Statistical Analysis

The experimental results were obtained from three biological replicates (*n* = 3) and are shown as mean values with associated standard deviations (SD). Statistical analyses were performed utilizing the R software platform (Version 1.6.2). A one‐way analysis of variance (ANOVA) was employed to identify differences among treatment groups, with *p* values below 0.05 considered statistically significant. Furthermore, multivariate techniques, specifically PCA and Orthogonal Partial Least Squares Discriminant Analysis (OPLS‐DA), were utilized to investigate significant metabolic alterations and categorization trends among groups. The OPLS‐DA model underwent validation with a seven‐cycle cross‐validation and permutation test, affirming its dependability and predictive capability. We identified notable discriminative metabolites using Variable Importance in Projection (VIP) scores exceeding 1 and *p* values below 0.05 from Student's *t*‐tests, deemed statistically significant. These thresholds were employed to discern the most pertinent metabolites accountable for group disparities. The distinct delineation of groups illustrated in the PCA and OPLS‐DA score plots further substantiated the statistical significance of the treatment effects. For pathway‐level analysis, notable metabolites were linked to the KEGG database (http://www.genome.jp/kegg/). Pathways with adjusted *p* values below 0.05 were considered significantly enriched. We employed the Stats module from the SciPy Python library (https://docs.scipy.org/doc/scipy/) to ascertain statistical significance in enrichment analysis. It enhanced our confidence in the accuracy of the biological significance of route alterations.

## 3 Results

### 3.1 Whole Lipid Classification Annotation

**FIGURE 1:**
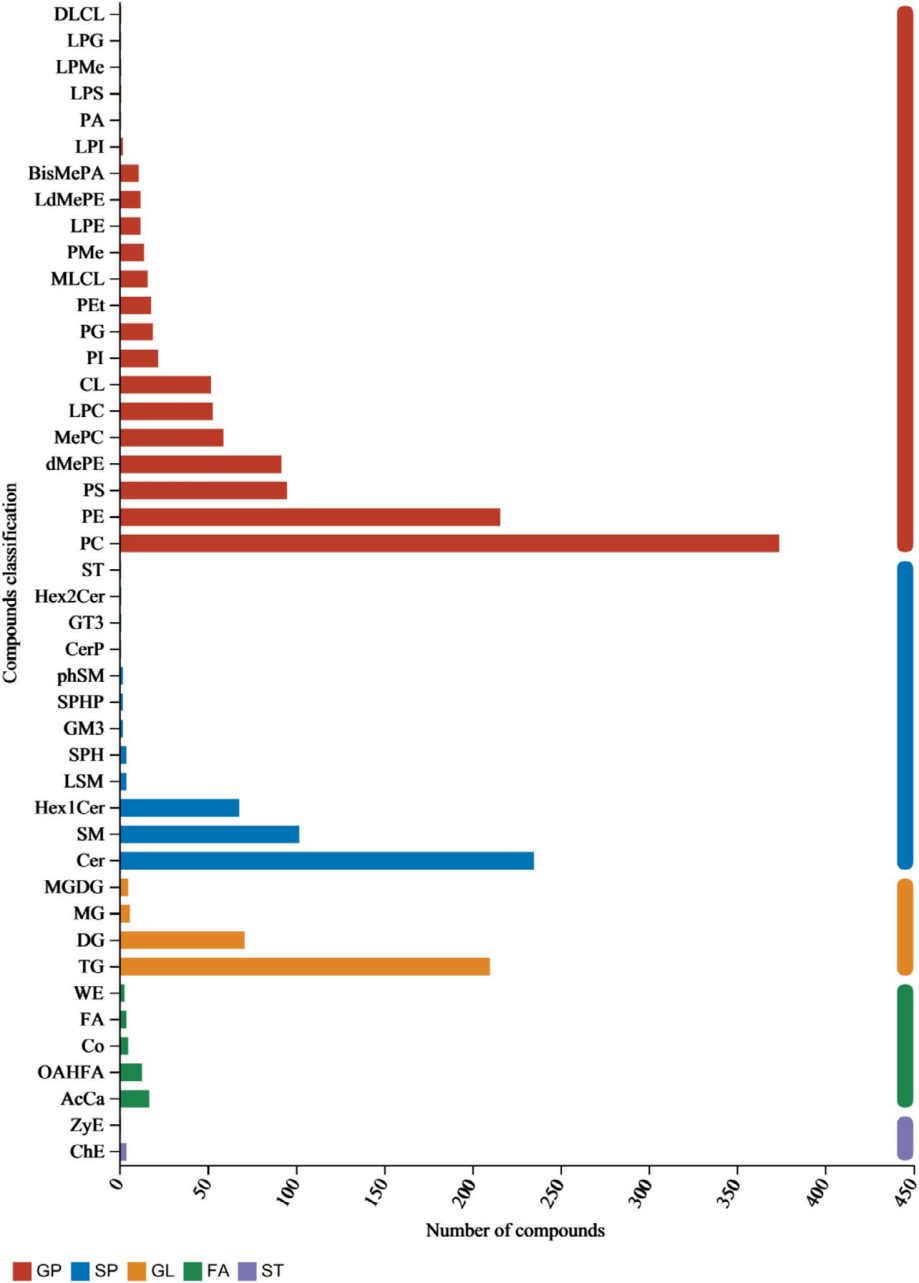
Whole lipid classification annotation. FA, fatty acyl glycerides; GL, glycerides; GP, glycerophospholipids; SP, sphingolipids; ST, sterol.

Whole lipid classification annotation results anticipated that 1310 lipid species were classified into 32 distinct lipid classes. The top 10 lipid classes listed in descending order are 374 (28.55%) phosphatidylcholines (PCs), 216 (16.49%) phosphatidylethanolamines (PEs), 210 (16.03%) triacylglycerols (TGs), 102 (7.79%) sphingomyelins (SMs), 95 (7.25%) phosphatidylserines (PSs), 68 (5.19%) hexosylceramides (Hex1Cer), 59 (4.50%) monomethyl‐PCs (MePCs), 53 (4.04%) lysophosphatidylcholines (LPCs), 52 (3.96%) dimethyl‐PCs (dMePCs), and 22 (1.68%) phosphatidylinositols (PIs) (Figure 1).

### 3.2 Differential Metabolites Analysis

**FIGURE 2:**
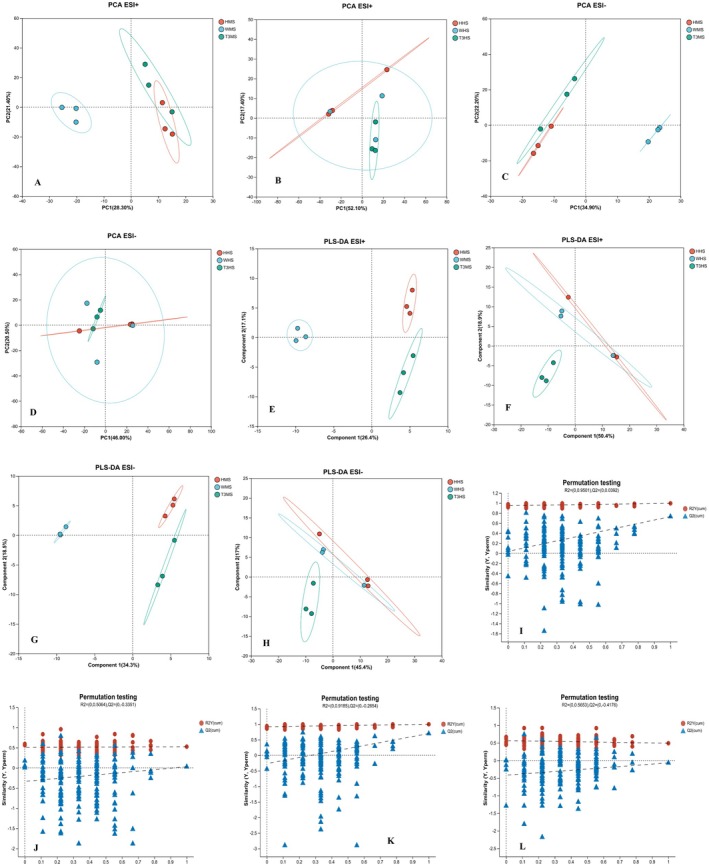
Differential metabolites analysis: PCA (A–D) and PLS‐DA (E–L) of the lipidomic data obtained from Huang, Wei, and T3BS muscle and head samples. HHS, Huang head sample; HMS, Huang muscle sample; T3HS, T3BS head sample; T3MS, T3BS muscle sample; WHS, Wei head sample; WMS, Wei muscle sample.

The PCA and PLS‐DA lipidomic profiles for the muscle and head segments of *L. vannamei* in the Huang, Wei, and T3BS groups demonstrated distinct clustering and differentiation among the treatment groups. The QC samples were tightly clustered, indicating the reliability of the MS analysis approach (Figure 2A–D). The PLS‐DA score plots exhibited clear differences among the treatment groups with effective model performance metrics (R^2^X, R^2^Y, and Q^2^), validating the models' excellent discriminative and predictive abilities (Figure 2E–H). For muscle tissues, the PLS‐DA models recorded R^2^X values of 0.264 (ESI+) and 0.343 (ESI−), R^2^Y values of 0.497 (ESI+) and 0.495 (ESI−), along with Q^2^ values of 0.313 (ESI+) and 0.352 (ESI−), demonstrating strong predictive capabilities. In contrast, for head tissues, the models showed R^2^X values of 0.504 (ESI+) and 0.454 (ESI−), R^2^Y values of 0.279 (ESI+) and 0.246 (ESI−), and Q^2^ values of 0.009 (ESI+) and −0.091 (ESI−), reflecting a lower predictive performance in comparison to muscle tissues (Figure 2I–L). The variable importance in projection (VIP) values obtained from the PLS‐DA models (VIP ≥ 1) and statistical *p* values (*p* ≤ 0.05) were utilized to identify 120 differentially abundant lipids (DALs) regarded as potential biomarkers. These DALs showed considerable differences among the Huang, Wei, and T3BS groups, indicating the effects of *L. lactis* , salinity, and DO on the lipid profile (Akram et al. 2022).

### 3.3 Relative Abundance of Glycerophospholipid Metabolites

**FIGURE 3:**
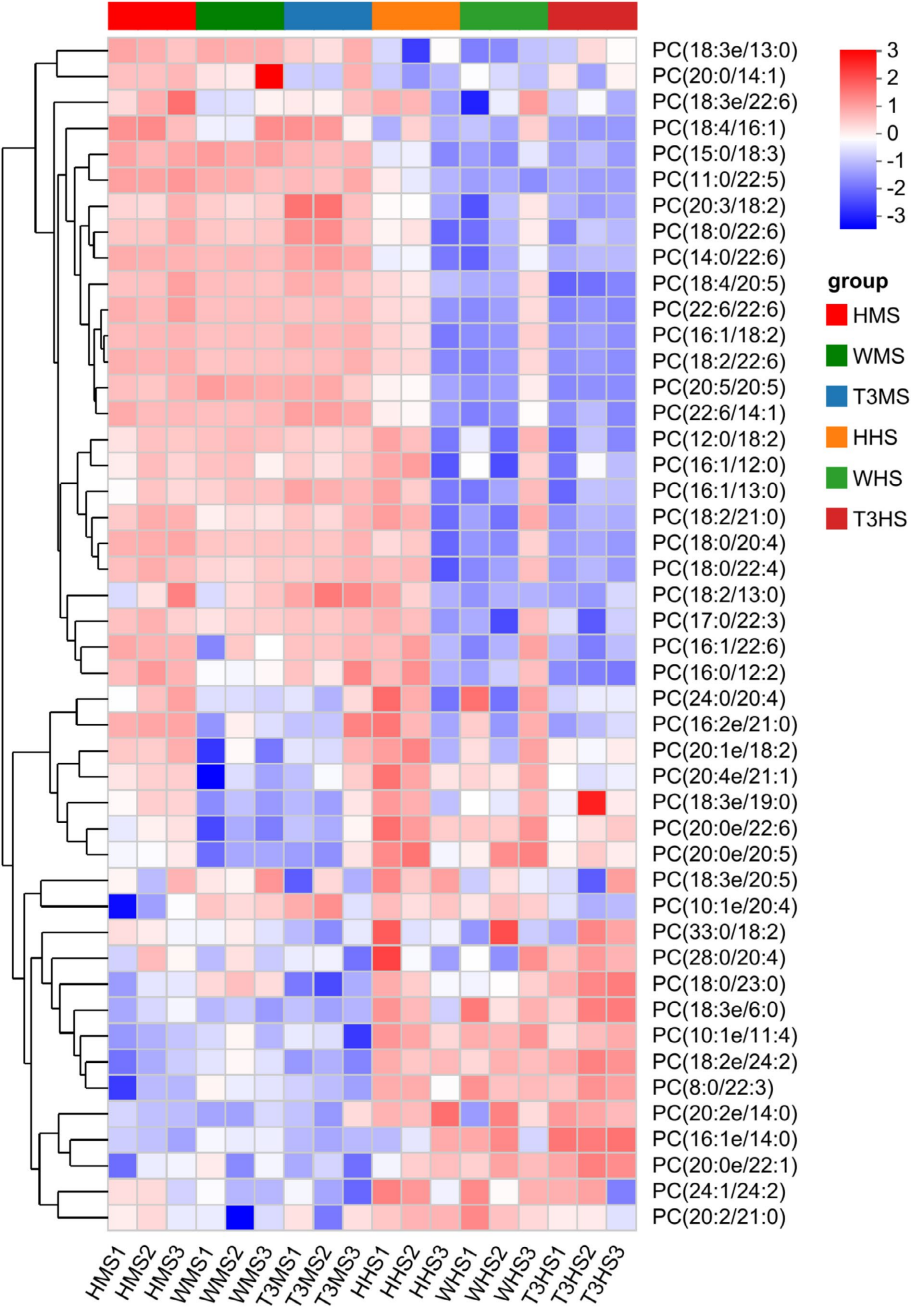
Relative abundance of glycerophospholipid metabolites. HHS, Huang head sample; HMS, Huang muscle sample; T3HS, T3BS head sample; T3MS, T3BS muscle sample; WHS, Wei head sample; WMS, Wei muscle sample. Each column in the figure represents a sample, and each row represents a metabolite.

The findings of glycerophospholipid among the Wei, Huang, and T3BS muscle and head treatment groups of *L. vannamei* exhibited significantly (*p* < 0.05) the highest abundance of glycerophospholipids such as PC (12:0/18:2), PC (18:0/20:4), PC (16:1/22:6), PC (10:1e/20:4) and PC (33:0/18:2) in the Huang head group followed by the T3BS head sample and Wei head sample. However, the data reported a nonsignificant (*p* < 0.05) difference in the muscle portions of all treatment groups (Figure 3).

### 3.4 Relative Abundance of Sphingolipid Metabolites

**FIGURE 4:**
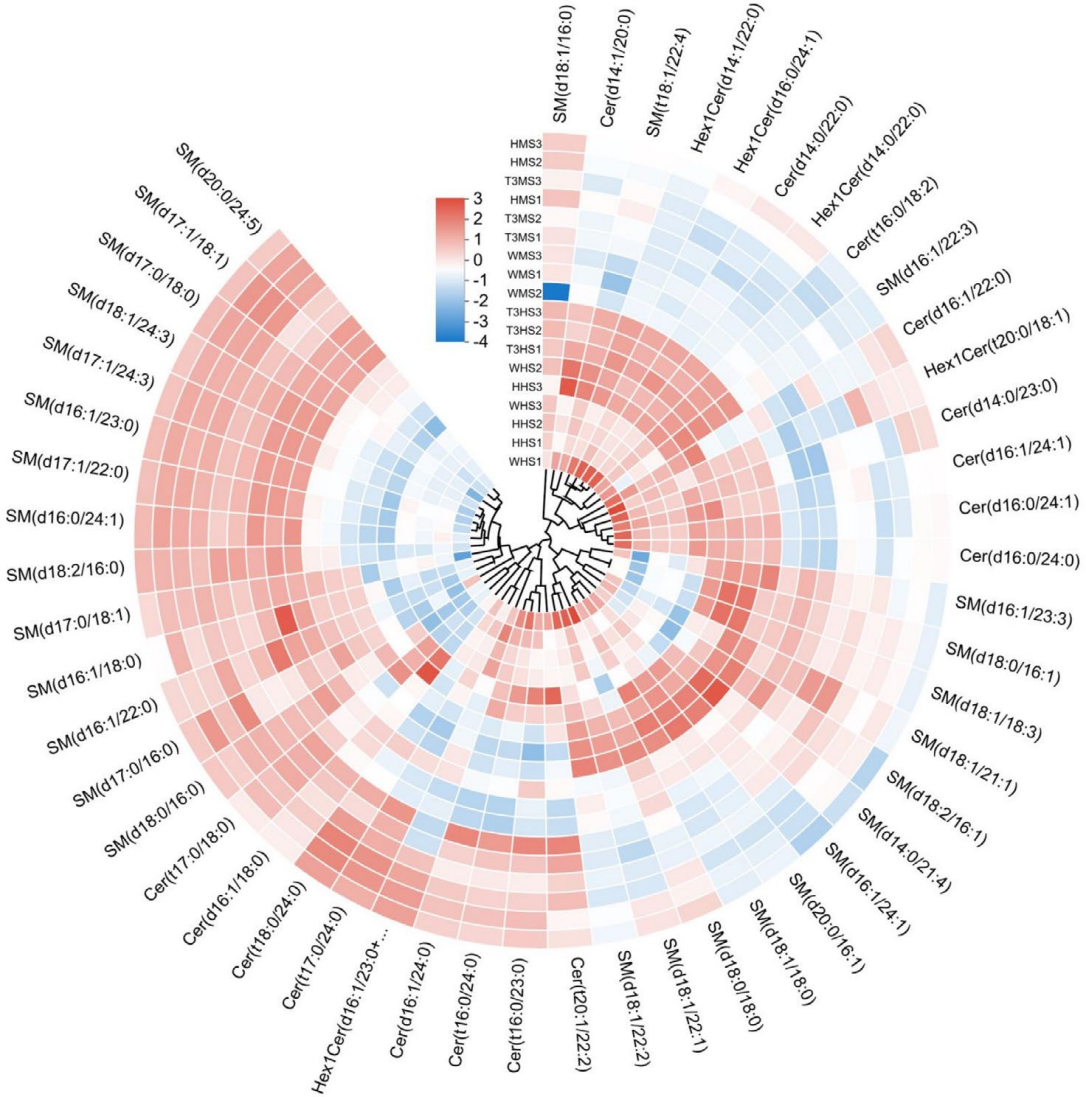
Relative abundance of sphingolipid metabolites. HHS, Huang head sample; HMS, Huang muscle sample; T3HS, T3BS head sample; T3MS, T3BS muscle sample; WHS, Wei head sample; WMS, Wei muscle sample. The name of the metabolite is on the outer circle of the figure, and the name of the corresponding group is on the cross‐section, and the color in the figure indicates the relative content of the metabolite in the corresponding groups.

The findings of sphingolipids among the Wei, Huang, and T3BS muscle and head treatment groups of *L. vannamei* exhibited significantly (*p* < 0.05) the highest abundance of sphingolipids such as (d14:1/20:0), SM (t18:1/22:4), SM (d18:0/16:1), SM (d18:1/18:3), SM (d14:0/21:4), SM (d16:1/24:1), SM (d20:0/16:1), and SM (d18:1/18:0) observed in the T3BS head samples followed by the Huang head and Wei head samples. At the same time, data showed nonsignificant (*p* < 0.05) differences among muscle parts of all three treatment groups (Figure 4).

### 3.5 Relative Abundance of Sterol Metabolites

The findings of sterols among the Wei, Huang and T3BS muscle and head treatment groups of *L. vannamei* exhibited significantly (*p* < 0.05) the highest abundance of sterols such as ChE (0:0), ChE (22:6), ST (m20:0/16:1), and ZyE (0:0) recorded in the Huang head sample, that is, 8.00, 7.97, 7.94, and 7.70, respectively, when compared to the T3BS head sample, that is, 7.21, 6.90, 7.51, and 6.34, and the Wei head sample, that is, 7.38, 7.13, 7.63, and 6.09, respectively. At the same time, data showed nonsignificant (*p* < 0.05) differences among muscle parts of all three treatment groups, respectively (Table 1).

### 3.6 Relative Abundance of Saturated Fatty Acid Metabolites

The analysis of saturated fatty acids in the Wei, Huang, and T3BS muscle and head treatment groups of *L. vannamei* revealed a statistically significant (*p* < 0.05) predominance of saturated fatty acids, including stearic acid, butyric acid, lauric acid, myristic acid, and arachidic acid, with the T3BS head samples exhibiting the highest concentrations: 5.34, 5.92, 4.73, 5.21, and 4.51, respectively. This was followed by the Huang head samples with values of 5.15, 5.02, 5.59, 4.98, and 3.54, and the Wei head samples with concentrations of 4.93, 4.62, 4.29, 3.79, and 4.22. Simultaneously, data indicated nonsignificant (*p* < 0.05) disparities among muscle regions across all three treatment groups (Table 2).

**TABLE 2:** Relative abundance of saturated fatty acid metabolites.

Fatty acids	HMS	WMS	T3MS	HHS	WHS	T3HS
Palmitic acid	0.03 ± 0.00^a^	0.02 ± 0.00^a^	0.02 ± 0.00^a^	5.46 ± 0.06^a^	5.20 ± 0.04^b^	5.46 ± 0.06^a^
Stearic acid	0.08 ± 0.00^a^	0.06 ± 0.00^a^	0.08 ± 0.00^a^	5.15 ± 0.31^b^	4.93 ± 0.03^c^	5.34 ± 0.12^a^
Butyric acid	1.09 ± 0.00^a^	1.10 ± 0.00^a^	1.10 ± 0.00^a^	5.02 ± 0.41^b^	4.62 ± 0.03^c^	5.92 ± 0.14^a^
Lauric acid	1.03 ± 0.00^a^	1.05 ± 0.00^a^	1.02 ± 0.00^a^	5.59 ± 0.31^a^	4.29 ± 0.05^c^	4.73 ± 0.12^b^
Myristic acid	1.20 ± 0.02^a^	1.19 ± 0.02^a^	1.21 ± 0.02^a^	4.98 ± 0.46^b^	3.79 ± 0.13^c^	5.21 ± 0.11^a^
Arachidic acid	0.92 ± 0.00^a^	0.91 ± 0.00^a^	0.92 ± 0.00^a^	3.54 ± 0.32^c^	4.22 ± 0.04^b^	4.51 ± 0.05^a^
Behenic acid	1.73 ± 0.03^a^	1.76 ± 0.03^a^	1.73 ± 0.03^a^	4.09 ± 0.42^a^	3.43 ± 0.97^b^	4.11 ± 0.42^a^

*Note:* HMS = Huang muscle sample, WMS = Wei muscle sample, T3MS = T3BS muscle sample. Results are presented as means ± SD and *n* = 3. Values with different alphabets differ significantly (*p* < 0.05) among HMS, WMS, and T3MS. HHS = Huang head sample, WHS = Wei head sample, T3HS = T3BS head sample. Results are presented as means ± SD and *n* = 3. Values with different alphabets differ significantly (*p* < 0.05) among HHS, WHS, and T3HS.

### 3.7 KEGG Pathway Enrichment Analysis

**FIGURE 5:**
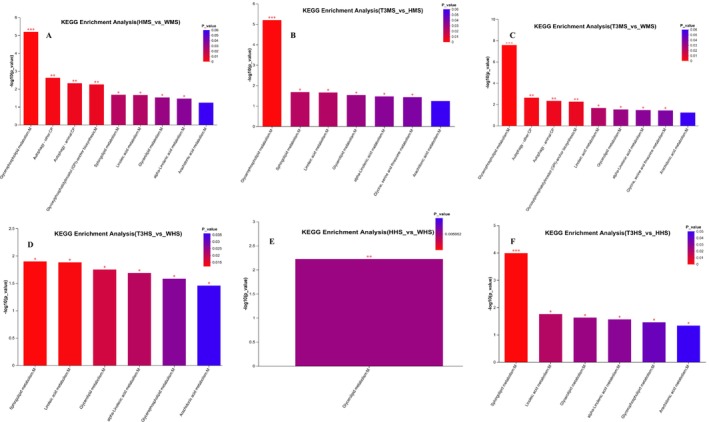
KEGG pathway enrichment analysis. HHS, Huang head sample; T3HS, T3BS head sample; T3MS, T3BS muscle sample; WHS, Wei head sample; WMS, Wei muscle sample.

The findings of KEGG pathway analysis among the Wei, Huang, and T3BS muscle and head treatment groups of *L. vannamei* exhibited significantly (*p* < 0.05) high enrichment of glycerophospholipids, sphingolipids, glycerolipids, linolenic and alpha‐linolenic acids metabolism pathways in T3BS muscle and head groups, respectively, among the top 20 KEGG enrichment pathways (Figure 5A–F).

## 4 Discussion

Among the whole lipid classification annotation, PCs and PEs are the predominant glycerophospholipids essential for crucial roles in sustaining cell membranes' structural integrity and permeability (Bandu et al. 2018; Krishnaveni et al. 2023). These results align with the earlier research by Yu et al. (2020), wherein the authors demonstrated the PCs and PEs as predominant phospholipids in *Penaeus kerathurus* and *Squilla mantis* . Sphingolipids are essential for maintaining membrane fluidity and lipid signaling, as exemplified by SM and Hex1Cer. Sun et al. (2020) demystified that SM is a phosphosphingolipid of major importance in muscle membranes and was found in high concentrations in white‐leg shrimps and crustaceans. Triglycerides form a significant element of the lipid profile, highlighting their crucial roles in energy reserves and intricate metabolism management (Jannathulla et al. 2022). Muscle tissue contributes only to movement and energy reserve, requiring saturated fatty acids and triacylglycerols (TGs) to act as energy reserves. This corresponds with the findings of Yuan et al. (2022), who explained shrimp muscular tissues emphasizing fatty acid metabolism as an energy source, thus justifying the high content of saturated fatty acids that this study's muscle samples witnessed. Conversely, the cephalothorax in the shrimps is vital in immune responses, neuroendocrine communication, and the metabolism of nutrients. This is why there are more glycerophospholipids and sphingolipids in the skull. Such lipids are important in stabilizing membranes and signal conduction, particularly in neurons and immune cells (Fajardo et al. 2022). The variations in lipid profiles can be attributed to the distinct metabolic functions of various organs. The variation in lipid profiles arises from the unique metabolic functions of various organs. Muscle tissue plays a key role in energy storage and lipid metabolism for the tissue to have a high percentage of saturated fatty acids. The immunological function, neurotransmission, and stress adaptation of brain tissue rely on glycerophospholipids and sphingolipids for the tissue to maintain cell signaling pathways and fluidity of the cell membrane (Wu et al. 2016). Another study Amin (2018) discovered that probiotic‐supplemented mollusks had enhanced head phospholipid content. This is a testimony to the critical role of the brain in immune response and stress control. Due to its central role in immunoregulation, neurotransmission, and stress response, the head was chosen for lipidomics. Chen et al. (2014) hypothesize that lipids in the cranial region play a crucial role in maintaining cell membrane integrity and the immune system, which are essential for survival in the face of changes such as a change in salt or dissolved oxygen content. For improving shrimp resilience in aquaculture, lipidomics research on the head informs how probiotics change lipid profiles associated with immune health and stress tolerance (Sidira et al. 2024).

Phosphatidylcholine (PC), a phospholipid found in cell membranes, is the primary lipid constituent of crustaceans' hemolymph lipoprotein transportation system (Zhang et al. 2019). Inoculation of *L. lactis* D1813 stimulated lipid metabolism by enhancing enzymatic activity associated with glycerophospholipid synthesis and nutrient absorption (Hu et al. 2024). Similarly, Huan et al. (2020) found that probiotics improve digestion and lipid profiles, while glycerophospholipids and immune‐related pathways were listed in higher numbers. Similarly, Liang et al. (2022) demonstrated that inoculation of *Bacillus subtilis* in *Penaeus modem* at a salinity of 20–25 ppt promoted the lipid metabolism pathways and showed downregulation in glycerophospholipids and also lowered oxidative stress. Similarly, Goh et al. (2022) showed a marked enhancement in glycerophospholipid abundance, lower cholesterol, and triglyceride synthesis on exposure of zebrafish larvae to *L. acidophilus* . Also, Zhang et al. (2024) indicated that glycerophospholipid production is closely linked to the activation of hypoxia‐inducible factor 1 genes, which facilitate membrane remodeling and stress adaptation. *L. lactis* enhanced feed efficiency, regulated immunological processes and improved the glycerophospholipids abundance in white leg shrimp (Adil et al. 2025). A study by Li et al. (2023) explained that inoculation of *L. lactis* WFLU12 with *Paralichythys olivaceus* diet with experimental conditions of 10 ppt salinity and 7.5 mg/L DO level improved the relative abundance of glycerophospholipids. Likewise, Q. Yu et al. (2025) demonstrated that low DO levels (2.5 mg/L) and elevated salinity conditions (35 ppt) cause hypoxia in shrimp, which hinders the activity of enzymes essential for lipid biosynthesis, including acetyl‐CoA carboxylase and fatty acid synthase, resulting in diminished synthesis of glycerophospholipids. Michael and Koshio (2016), inoculation of *Vibrio parahaemolyticus* with *M. japonicus* at 15 ppt salinity and 7.8 mg/L DO delineates higher abundance in phospholipids and also portrayed higher survival rates of 81% on exposure to 24 h freshwater conditions. Dietary feeding of live or freeze‐dried *L. lactis* to white leg shrimp reduced the vibrios in the culture water and the hepatopancreas. The intestinal tract of tiger shrimps showed the glycerophospholipid profile enrichment (Baharuddin et al. 2024). Another study by Corral‐Ricque et al. (2019) reported that mollusks inoculated with *L. lactis* showed a higher abundance of phospholipids, especially when DO levels were higher than 7.5 mg/L, which improved cellular membrane integrity and lipid metabolism. Another study by Shen et al. (2023) demonstrated that the addition of probiotics with an increase in salinity over 30 ppt, coupled with low DO levels (2 mg/L), affected the osmoregulatory capacity of *L. vannamei* larvae, leading to disturbances in energy metabolism, which reduced TCA cycle activities, glycerophospholipid and glycine abundance.

Sphingolipid abundance increased significantly in zebrafish fed the transgenic phytase‐expressing probiotic *Bacillus subtilis* , which improved cellular signaling, membrane integrity, and lipid metabolism (Duan et al. 2021). Another study by X. Zheng et al. (2022) showed that *L. lactis* D1813 probiotic upregulated sphingomyelin levels and lowered oxidative stress, aiding cellular adaptation to high salinity and low DO. Ringø et al. (2022) indicated that probiotics improve sphingolipid metabolism, improve the stress resistance of cell membranes, and increase shrimp resilience at high salinity and DO (30 ppt, 8.5 mg/L). Adding *L. lactis* , a potent probiotic, to shrimp significantly improved their flavor. It led to a dramatic rise in compounds associated with the biosynthesis of unsaturated fatty acids, arachidonic acid, amino acids, and sphingolipid metabolites at a salinity level of 25 ppt. The addition of *L. lactis* , a powerful probiotic, to shrimp greatly improved their flavor and led to a dramatic rise in compounds associated with the biosynthesis of unsaturated fatty acids, arachidonic acid, amino acids, and sphingolipid metabolites at a salinity level of 25 ppt (Goh et al. 2023). A study by Kojima et al. (2010) showed a substantial (*p* < 0.05) increase in the levels of sphingomyelin and ceramide in *Penaeus vannamei* upon the addition of *L. lactis* at salinity and DO concentrations of 25 ppt and 4 mg/L, respectively. Similarly, Liang et al. (2022) showed that by increasing sphingolipid levels, *Lactobacillus rhamnosus* improved the mollusk's lipid transport systems and osmoregulation when given to them in high salinity (25 ppt) and DO (6.8 mg/L) conditions. Another study by Zhao et al. (2022) indicated a 30% decrease in sphingolipid abundance in *L. vannamei* at salinity levels of 5 ppt and DO levels of 3.5 mg/L due to a lack of osmotic stress. Grass carp ( *Ctenopharyngodon idella* ) fed a *L. plantarum* diet showed considerable enhancement in lipid metabolism through manipulation of the gut microbiota, resulting in elevated levels of sphingolipids (Tian et al. 2022). The hindgut microbiota of grass carp fed fish meal (FM) and Sudan grass ( *Sorghum sudanense* ) changed quickly when *Bacillus subtilis* was added at 7 mg/L dissolved oxygen in 11 days. Nonetheless, genes related to sphingolipid metabolism exhibited no significant alterations due to the dietary modification (Hao et al. 2017). Similarly, Sun et al. (2020) observed that *Marsupenaeus japonicus* salinity (> 10 ppt) inoculation of *Lactobacillus reuteri* reduced abundance by modulating sphingolipid metabolic pathways. Another study by Y. Zhang et al. (2020) noticed a decrease in sphingolipid amount in *Marsupenaeus japonicus* when subjected to low salinity (2 ppt) due to osmotic stress caused by *B. subtilis* . Raghuvaran et al. (2023) found that injecting *Penaeus monodon* with *Lactobacillus salivarius* at a DO concentration of 2 mg/L reduced sphingolipid concentrations through interference with membrane integrity and lipid metabolism.

The Huang group exhibited a greater concentration of sterols because they supplemented with *L. lactis* D1813, a strain that enhances sterol synthesis by reducing oxidative stress and improving lipid absorption (Lina Liu et al. 2023). Likewise, K. Chen et al. (2014) found that shrimp with greater sterol levels are better equipped to deal with osmotic stress, ultimately benefiting their survival and growth. Another study by Vivekanandan et al. (2009) showed that the quantities of sterols, specifically ChE (0:0) (8.21 mg/L) and ChE (22:6) (7.90 mg/L), were reduced in *Penaeus monodon* treated with *Bacillus subtilis* at salinity and DO levels of 10 ppt and 8 mg/L, respectively. Similarly, Dhanasiri et al. (2023) reported that feeding *Pediococcus acidilactici* to Atlantic salmon in water with a moderate salinity level (24 ppt) and 6.5 mg/L DO increased the amount of sterols such as ChE (0) and ChE (22:6) by 7.85 and 7.92, respectively. Ma et al. (2024) research stated that *Portunus trituberculatus* exhibited reduced levels of sterols, such as ChE (0:0) at 6.23 and ChE (22:6) at 6.75, when the salinity was less than 35 ppt and the DO was 2 mg/L. While Raju and Benjakul (2022) showed that adding *L. lactis* to *Litopenaeus setiferus* at salinity levels of 10 ppt and DO levels of 6.5 mg/L significantly increased the sterol levels of ChE (0:0) and ChE (22:6), which were 7.95 and 7.72, respectively. A study by Saini et al. (2020) explained a significant increase in the level of sterol upon exposure of *L. vannamei* to *L. rhamnosus* under 8 ppt salinity and 7.5 mg/L DO.

The increase in saturated fatty acid levels in shrimp tissues can be linked to the breakdown of dietary fats by the probiotic effects (Yang et al. 2023). A retroactive study by Chen et al. (2020) illustrated that *Dicentrarchus labrax* supplementation with *L. lactis* at salinities of 14–30 ppt and DO of 7.5 mg/L led to a significant elevation of stearic acid (C18:0) and arachidic acid (C20:0). Enrichment of fatty acids and lipid metabolism genes in the allochthonous hindgut microbiota of Western Cascade rainbow trout was significant with *L. lactis* in comparison to the Eastern Cascade fish that did not receive probiotics, though the latter had a more diverse microbial population (Oleinikova et al. 2024; Yildirimer and Brown 2018). Likewise, in another research carried out by Yuan et al. (2025), it was elaborated that high salinity (> 25 ppt) and moderate concentrations of DO (> 6 mg/L) facilitated aerobic respiration, which increased abundance levels of lauric acid (C12:0) and myristic acid (C14:0) in fish. *L. bacillus* strains optimize fatty acid metabolism by promoting food absorption and stress tolerance (Du et al. 2022). *L. rhamnosus* inoculation in association with *Macrobrachium rosenbergii* at DO levels of 10 mg/L showed increased enzymatic activity, aerobic respiration, and metabolic activity of fatty acids, promoting the abundance level. Another related study conducted by Mozanzadeh et al. (2023) revealed that *Lates calcarifer* supplemented with *L. plantarum* at 12–35 ppt salinity and 3.1–6.8 mg/L DO concentrations retained varying quantities of butyric acid and stearic acid, that is, 4.21–3.81 and 5.34–4.19, respectively. With the rise in salinity, water loss occurs in cells, reducing stearic acid and arachidic acid production (Mahdhi et al. 2020). Similarly, Butt et al. (2021) showed that the inoculation of *Penaeus vannamei* with *Lactobacillus rhamnosus* under 10 ppt salinity and a DO level of 6.5 mg/L resulted in notably greater levels of stearic acid. Research carried out by Najmi et al. (2018) showed that the exposure of *P. vannamei* to *Bacillus subtilis* under 5 ppt salinity and 8 mg/L of DO resulted in a significant reduction of the myristic acid level, that is, 6.44, in comparison to freshwater *L. vannamei* , which contained 8.76 values. Elevated DO (> 7 mg/L) raised the amount of the enzyme enolase, which is necessary for the synthesis of saturated fatty acids (Pratama et al. 2023). The superior growth performance and reduced feed conversion ratio of white shrimps consuming *L. lactis* ‐supplemented diets may be attributed to the bacterium's capacity to enhance digestive enzyme activity, thereby augmenting feed digestibility and subsequently improving growth rates through increased availability of fatty acid metabolites (Adel et al. 2017).

The enrichment could be linked to *L. lactis* D1813 inculcation, moderate DO levels, and high salt concentrations, improving growth performance, immunity, and nutrient absorption (Shao et al. 2020). Likewise, *L. lactis* D1813 produces glycerophospholipids, improves cell protection, and enhances immunological responses (Wishart et al. 2007). High salinity (< 20 ppt) and moderate DO (10 mg/L) promote osmotic equilibrium and aerobic respiration, which enhances the metabolic functions of lipids (Zheng et al. 2021). Depleted oxygen levels (> 3.5 mg/L) impede the transformation and utilization of pyridoxine‐folates for lipid synthesis and cellular functions (Hu et al. 2024). Similarly, *L. lactis* improved the glycerophospholipids and sphingolipids metabolism in *L. vannamei* (Li et al. 2023; Shao et al. 2019; Wang et al. 2022). The transcriptions of genes involved in cholesterol (hnf4α and npc1l1) and triglyceride (fit2 and mgll) metabolism were downregulated in zebrafish larvae when *L. lactis* (IMC 501) in *L. vannamei* was administered. This resulted in minimal enrichment of the cholesterol and triglyceride pathways, but increased enrichment of the linolenic and alpha‐linolenic acids pathways (Ruan et al. 2021). Another study by Kong et al. (2024) reported that the administration of *B. subtilis* in zebrafish for 8 weeks of experimental design with 30 ppt salinity and 7.5 mg/L DO level significantly decreased the expression of sphingolipid and glycerolipid pathways. Another study by Du et al. (2019) also showed enrichment of glycerophospholipids with efficient ATP and energy production from aerobic metabolism helping enhance the biosynthetic pathways when *L. lactis is* inoculated with white shrimp at 35 ppt salinity. According to Huang et al. (2020), a gradual increase in salinity over 21 days with osmotic stress resulted in the upregulation of pathways involved in glycerophospholipid and sphingolipid biosynthesis due to the strain on cellular functions. Kangpanich (2016) explained that salinity levels of 20–25 ppt for 14 days significantly increased the linolenic and alpha‐linolenic acid metabolism pathways, which enhanced probiotic proliferation and reduced osmotic stress. Also, Simchovitz et al. (2017) found that the inoculation of *Lactobacillus rhamnosus* in *Penaeus vannamei* at high salinity of 35 ppt resulted in significant upsurge of glycerophospholipid abundance owing to HIF‐1 gene activation.

## 5 Conclusion

This study exhibited significant improvements in the lipidomic characterization of *L. vannamei* using UHPLC–MS/MS‐based lipid profiling under the influence of *L. lactis* D1813, increased salinity, and reduced DO levels. The findings indicated that the Huang group possessed the highest levels of glycerophospholipids and sterols, whereas the T3BS group exhibited a greater concentration of sphingolipids and saturated fatty acids. The KEGG pathway analysis indicated that the T3BS muscle and head exhibited a significantly higher abundance of glycerophospholipids, sphingolipids, linolenic acid, and alpha‐linolenic acid metabolic pathways. The administration of *L. lactis* D1813 at 10^6^ CFU/mL, in conjunction with 25 ppt salinity and 3.5 mg/L DO, significantly elevated the concentrations of sphingolipids, saturated fatty acids, and metabolites linked to linolenic and alpha‐linolenic acids in *L. vannamei* . *L. lactis* D1813's ability to modify tissue‐specific lipid profiles in response to environmental stressors may improve prawn resilience, stress tolerance, and survival rates in semi‐intensive and intensive aquaculture environments with fluctuating salinity and oxygen levels. From an aquaculture perspective, these findings suggest that the probiotic supplementation of *L. lactis* D1813 could effectively enhance farming practices, improve feed efficiency, and strengthen disease resistance in shrimps. This approach may enhance the sustainability and efficacy of shrimp farming by diminishing antibiotic reliance and improving shrimp health and performance under challenging conditions. Improving shrimp lipid profiles, particularly concerning bioactive lipids like phospholipids and omega‐3 precursors, offers potential benefits for human cardiovascular and cognitive health. The enhancements in shrimp quality are not the primary emphasis yet, but they may still hold significant market value, particularly among health‐conscious consumers seeking products with greater nutritional advantages. Further investigation is necessary to examine the therapeutic potential of lipids derived from *L. vannamei* concerning human health, specifically concerning cardiovascular health, cognitive function, and anti‐inflammatory effects.

## Author Contributions

**Felix Kwashie Madilo:** software (equal), writing – review and editing (equal). **Muhammad Adil:** data curation (equal), investigation (equal), methodology (equal), writing – original draft (equal). **Muhammad Talha Afraz:** methodology (equal), software (equal), writing – original draft (equal). **Muhammad Waseem:** resources (equal), supervision (equal), writing – review and editing (equal). **Muhammad Rizwan Javed:** methodology (equal), software (equal), writing – original draft (equal). **Muhammad Faisal Manzoor:** resources (equal), supervision (equal), writing – review and editing (equal). **Xinglong Xiao:** conceptualization (equal), methodology (equal), supervision (equal). **Basim M. Alohali:** funding acquisition (equal), writing – review and editing (equal). **Isam A. Mohamed Ahmed:** validation (equal), writing – review and editing (equal).

## Ethics Statement

This study was carried out per the recommendations of the Animal Ethics Committee of Guangdong Province, China.

## Conflicts of Interest

The authors declare no conflicts of interest.

## Data Availability

All lipidomic data is available in the Metabolome Cloud analysis Agronomy database under accession MJ20231007029. The data analysis pipeline of lipidomic analysis is available on GitHub at https://analysis.majorbio.com/dia4d/report.
